# Psychological Impact of COVID-19 on Dental Graduates: A Cross-Sectional Study

**DOI:** 10.7759/cureus.50673

**Published:** 2023-12-17

**Authors:** Mai Salah El-Din, Rahaf M Alhazmi, Rania Moussa

**Affiliations:** 1 Department of Prosthodontics, Alexandria University Main Hospital, Alexandria, EGY; 2 Department of Primary Healthcare-Dental Services, Ministry of National Guard - Health Affairs, Jeddah, SAU; 3 Department of Substitutive Dental Sciences, College of Dentistry, Taibah University, Al-Madinah Al-Munawwarah, SAU

**Keywords:** pandemic, gad-7 scale, covid-19, dental interns, online questionnaire

## Abstract

Background

The pandemic-induced physical closure of educational institutions led to challenges for senior dental students that might affect their psychological status. This study aimed to assess stress, anxiety, and subjective worries among dental interns post-COVID-19.

Methodology

A self-structured questionnaire was sent to Alexandria University and Taibah University dental interns. The questionnaire included sociodemographic data, self-reported comorbidities, behavioral risk factors, Kessler Psychological Distress Scale (K-10), Generalized Anxiety Disorder 7-Item (GAD-7) scale, and future subjective worries regarding the dental profession post-COVID-19.

Results

A total of 129 interns participated, with the majority (79.1%) being females. Overall, 90.7% were unmarried, 80.6% lived with a family, 93.8% were free from systemic comorbidities, 82.17% were satisfied with their current life, and 63.6% felt positive quite often. K-10 distress levels were reported as very high (38%) and high (33.3%), and perceived satisfaction with current social life was significantly associated with moderate-to-high distress levels. Feeling positive about life quite often was significantly associated with very high distress levels. Mild GAD-7 anxiety levels were reported in 40.3% and were significantly associated with females, who perceived positive satisfaction with their current social life and felt positive about life. Most agreed that their profession would be risky, their ability to perform their job to the best would be jeopardized, and they would need extra precautions in clinical training post-COVID-19; however, they disagree with postponing their training until COVID-19 cases declined entirely.

Conclusions

Dental interns were affected by the lockdown, especially female interns. Psychological therapy is advised for stressed dental students.

## Introduction

The coronavirus pandemic broke out worldwide by the end of 2019 and the beginning of 2020, causing a clinical disease known as COVID-19. COVID-19, transmitted through droplets and direct contact, rapidly spread worldwide to infect all age groups with high death rates. This fact forced the World Health Organization (WHO) to declare COVID-19 a pandemic in March 2020 [1].

Since then, although daily life has almost returned to normal, the WHO has acknowledged many variants of the COVID-19 virus (namely, Delta, Gamma, Beta, Alpha, and the currently circulating variant Omicron) [2]. All variants showed little changes in the structure; however, scientists reported changes in the characteristics, including how it transmits from one person to the other, the related illness severity, the efficiency of the vaccines, prescribed treatments, specific investigations, and any community health measures. The evolution of the variants indicated that current strategies and measures recommended by the WHO ought to continue to work in infection control to reduce virus transformations that have unfavorable public health effects [2].

The pandemic psychologically affected the general population due to the worldwide lockdown and the calls for social distancing. Healthcare workers, including dentists, have experienced additional burdens from direct contact with infected patients, increasing the chances of infection and carrying it to their families. Even worse, in some instances, society rejected them as a possible source of infection [3].

Considering how COVID-19 spreads, dental professionals need to be aware, given the high vulnerability to viruses spreading from their patients in face-to-face interaction and repeated exposure to saliva, blood, other body fluids, and bioaerosols generated during dental procedures. The Occupational Safety and Health Administration identified dental care specialists in the high-exposure threat group for COVID-19 [4].

Dental procedures place dental practitioners and students at higher risk of infection, thus increasing worries and possibly leading to stress, anxiety, and depression [5].

Dental education has been one of the practices adversely affected during the pandemic, and the conventional dental teaching models of person-to-person educational policy had to be partially replaced by digital or virtual setups to avoid the assembly of dental students in tight spaces. Several dental schools used virtual patient substitutes to avoid direct contact with actual patients, which might have reduced students’ chances to train suitably for their future careers [6].

Stress is a condition of mental or emotional adverse changes resulting from adverse or very demanding circumstances that demand exceeded adaptation than usual. Anxiety is a physiological reaction of the body to reduce or dominate a pending threat or danger. It is a feeling of strain, apprehensive thoughts, and body changes such as increased blood pressure. Depression is a state of emotional pain, unhappiness, sorrow, loss of concern or desire, or thoughts of guilt or low self-esteem that appear in response to an unpleasant event or situation [7].

Various findings reported that dental learners have psychological difficulties, including stress, anxiety, depression, and obsessive-compulsive disorders. The degree of psychological complications varied with gender, the level of study, whether they were in the preclinical or clinical phase, and with or without family support [8]. The psychological stress was perceived as low [9], moderate [10], high, and very high [11-13].

This study aimed to comparatively assess the degree of depression, anxiety, and stress of fresh graduate dental students in Egypt and Saudi Arabia post-COVID-19 pandemic after almost returning to their normal activities. In addition, it aimed to investigate the personal worries of fresh dental graduates regarding their future dental profession. The null hypothesis of this study is that the COVID-19 pandemic does not affect the stress, anxiety, and depression levels of dental interns in Egypt and Saudi Arabia.

## Materials and methods

This was an observational cross-sectional study conducted among fresh graduate dental interns at two universities in Egypt and Saudi Arabia between May and July 2022. This study followed the bioethical values for medical research on human beings outlined by the Declaration of Helsinki regarding confidentiality, freedom, respect, and non-maleficence. The ethical committee of Alexandria University approved the research protocol (IRB number: 00010556-IORG 0008839; ethics committee number: 0419-03/2022). The protocol was accepted by the Taibah University College of Dentistry Research Ethics Committee (reference number: TUCDREC/15032022/RMoussa). These approvals were obtained before any research-related activities.

The sample size was computed using special software (Epi Info software, Centers for Disease Control and Prevention) based on an 80% power of the study, an expected incidence of 70% psychological distress based on a previous study from Saudia Arabia [3], and a confidence level of 95%. The estimated minimum sample size was 122.

Inclusion criteria were dental interns doing their internship training at the Faculty of Dentistry, Alexandria University, or College of Dentistry, Taibah University, which was a one-year training program at both universities. Interns of any nationality were included. Interns who graduated from the aforementioned universities or any other university were included. Dental interns under medications for systemic diseases, not willing to participate, and receiving psychological treatment (mental or behavioral rehabilitation, drug, or combination) were excluded.

The questionnaire was designed using Google Forms after referring to similar studies reported in the literature [10,14]. The questionnaire comprised 35 close-ended questions that needed around 10 minutes to be responded to by the applicants. The researchers contacted the target participants through emails and social networks. The questionnaire included an introductory page explaining the study type and objectives. The questionnaire included verification to certify that all the information was anonymous, provided a confidentiality assurance, and enclosed informed consent to participate in the study. Everyone could reject the evaluation if they did not desire to complete it. Only the investigators had access to the data and did not require personal details such as telephone number, full name, or address.

The questionnaire comprised four sections. The first section collected sociodemographic data, self-reported comorbidities, and behavioral risk factors. The second section assessed the psychological impact of COVID-19 using the Kessler Psychological Distress Scale (K-10). K-10 comprised 10 questions rated on a five-point Likert scale (none, a little, sometimes, most of the time, all the time) and scored from 1 (none) to 5 (all the time). All items were scored, and the total score for each participant was categorized into low (score 10-15), moderate (score 16-21), high (score 22-29), and very high (score 30-50) [14].

The third section included the Generalized Anxiety Disorder 7-item (GAD-7) scale that comprised seven questions about the level of anxiety experienced over the years of study during the COVID-19 outbreak. Each question was graded on a four-point scale (not sure, for two to three days, more than half of the days, and nearly every day) and scored from 0 (not sure) to 4 (nearly every day). Total scores were categorized into minimal, mild, moderate, and severe anxiety, where 0-4 was considered as none to minimal anxiety, 5-9 as mild anxiety, 10-14 as moderate anxiety, and 15-21 as severe anxiety [11].

The last section included seven questions about personal worries for the future dental profession after COVID-19 with a yes or no choice and the rating of the participant’s worries on a scale from 1 to 10 (where one was not worried at all and 10 was extremely worried) [11].

SPSS version 28 (IBM Corp., Armonk, NY, USA) was used for statistical analysis. Descriptive analyses were performed where categorical variables were reported as proportions and continuous variables as mean ± standard deviation (SD). The levels of psychological distress and anxiety were reported. Pearson’s chi-square test assessed the association between the observed distress, anxiety, personal worries, and sociodemographic variables. All statistical tests were done with a significance level of 5% (p < 0.05).

## Results

A total of 136 participants responded to the questionnaire, and 129 consented to participate, with a response rate of 94.85%. The participants belonged to the age range of 22-28 years. The predominant gender was female (79.1%). Most participants were unmarried (90.7%), lived with a family member (80.6%), and felt perceived safety in the living place concerning COVID-19 (88.4%). Most lived in Egypt (86.8%) and studied at Alexandria University (76.7%). Systemically, most were free from any comorbidity (93.8%), never smoked (88.4%), were satisfied with their current social life (82.17%), and quite often felt optimistic about life (63.6%). Table 1 shows the sociodemographic data of the population under study.

**Table 1:** Characteristics of the study population.

Characteristics	Subcategories	Total, n (%)
Age in years	Mean (± SD)	24.12 (±0.96)
	Range	22–28
Gender	Total	129
	Male	27 (20.9)
	Female	102 (79.1)
Marital Status	Total	129 (100%)
	Married	12 (9.3%)
	Unmarried	117 (90.7%)
	Divorced	-
	Widowed	-
Living status	Total	129 (100%)
	Live alone	10 (7.8%)
	With Family member	104 (80.6%)
	In shared house	15 (11.6%)
	In hostel	-
Perception of safety in the living place in relation to COVID-19	Total	129 (100%)
	Unsafe	15 (11.6%)
	Safe	114 (88.4%)
Country where you take your internship	Total	129 (100%)
	Egypt	112 (86.8%)
	Saudi Arabia	17 (13.2%)
Study University	Total	129 (100%)
	Alexandria University	99 (76.7%)
	Benghazi University	1 (0.8%)
	International Humanitarian University	1 (0.8%)
	International Medical University in Odessa Ukraine	1 (0.8%)
	International University of Africa	1 (0.8%)
	Kafr Elshikh	1 (0.8%)
	KAU	3 (2.3%)
	Kharkiv National Medical University	1 (0.8%)
	Khartoum	1 (0.8%)
	National University	4 (3.1%)
	Pharos University	1 (0.8%)
	Sudan International University	1 (0.8%)
	Taibah University	14 (10.9%
Comorbidities	Total	129 (100%)
	None	121 (93.8%)
	Diabetes	1 (0.8)
	Hypertension	-
	Kidney disease	-
	Heart disease	-
	Others	7 (5.4%)
Smoking status	Total	129 (100%)
	Smoker	12 (9.3%)
	Quitted smoking	3 (2.3%)
	Never smoked	114 (88.4%)
Perceived current social life	Total	129 (100%)
	Dissatisfied	23 (17.8%)
	Satisfied	106 (82.17%)
Feel positive about life	Total	129 (100%)
	Always positive	39 (30.2%)
	Quite often	82 (63.6%)
	Never	8 (6.2%)

**Figure 1:**
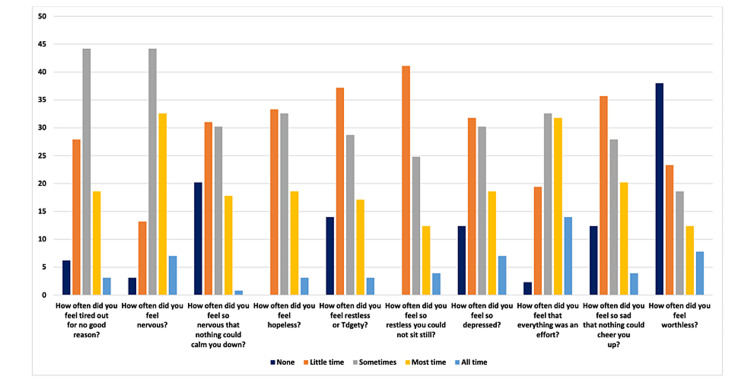
Bar chart of the responses to the psychological distress level among the study population (K-10) items. K-10 = Kessler Psychological Distress Scale

The summary of the participants’ responses to the psychological impact of COVID-19 using the K-10 is presented in Figure 1.

**Figure 2:**
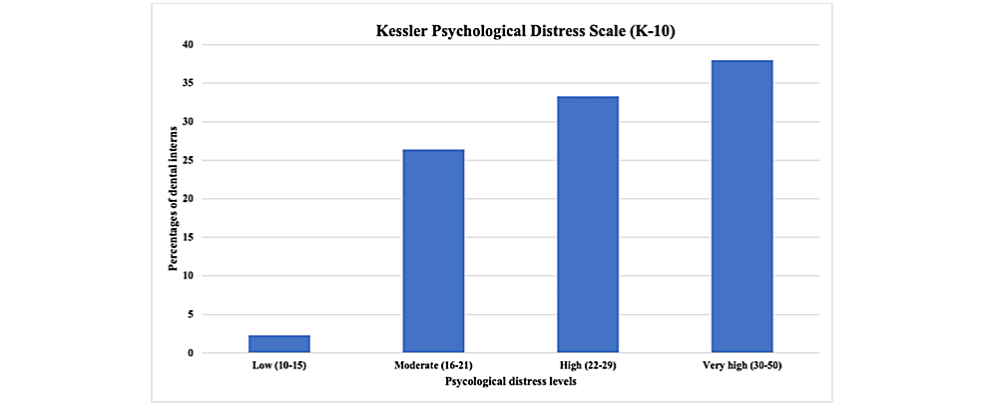
Bar chart of percentages of psychological distress levels among dental interns according to the K-10 scale. K-10 = Kessler Psychological Distress Scale

The total score for each participant was categorized, and the higher percentage was in the very high category (38%) and high (33.3%) psychological distress levels, as presented in Table 2 and Figure 2.

**Table 2:** Levels of psychological distress (K-10) categories. K-10 = Kessler Psychological Distress Scale

Severity level	Low	Moderate	High	Very high	Total	
Frequency	3	34	43	49	129	Mean ± SD = 27.36 ± 7.69
Percent	2.3	26.4	33.3	38	100	P-value = 0.006*

In addition, univariate analyses showed that noticed satisfaction with current social life was significantly associated with moderate-to-high distress levels. However, feeling optimistic about life quite often was significantly associated with very high levels of psychological distress compared to their counterparts (Table 3).

**Table 3:** Association of sociodemographic factors and psychological distress (K-10) levels. Based on Pearson’s chi-square test. P < 0.05 denotes a significant association. K-10 = Kessler Psychological Distress Scale

Variables	Category	Psychological distress (K-10) categories				Total	P-value
		Low	Moderate	High	Very High		
Gender	Male	2	9	9	7	27	0.177
	Female	1	25	34	42	102	
Marital status	Married	0	1	6	5	12	0.287
	Unmarried	3	33	37	44	117	
Living status	Alone	1	0	3	6	10	0.06
	With family	2	30	32	40	104	
	In shared house	0	4	8	3	15	
Perceived safety with COVID-19	Safe	3	31	38	42	114	0.72
	Unsafe	0	3	5	7	15	
Current country of residence	Egypt	3	31	36	42	139	0.606
	Saudi Arabia	0	3	7	7	17	
Systemic comorbidities	With systemic condition	0	2	2	3	7	0.931
	Free from any systemic condition	3	32	41	66	122	
Smoking status	Non-smoker	2	32	37	43	114	0.348
	Smoker	0	2	5	5	12	
	Quitted	1	0	1	1	3	
Perceived current social life	Satisfied	3	34	38	31	106	0.001*
	Dissatisfied	0	0	5	18	23	
Feel positive about life	Always positive	3	16	13	7	39	0.001*
	Never	0	0	0	8	8	
	Quite often	0	18	30	34	82	

**Figure 3:**
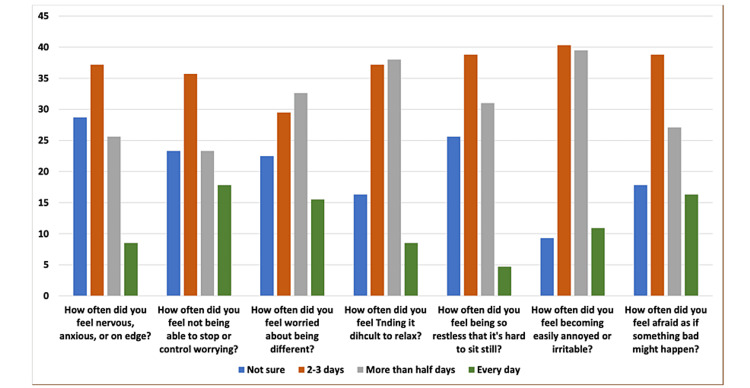
Bar chart of the anxiety level GAD-7 items among the study population. GAD-7 = Generalized Anxiety Disorder 7-Item

The participants’ responses to the GAD-7 scale are presented in Figure 3.

**Figure 4:**
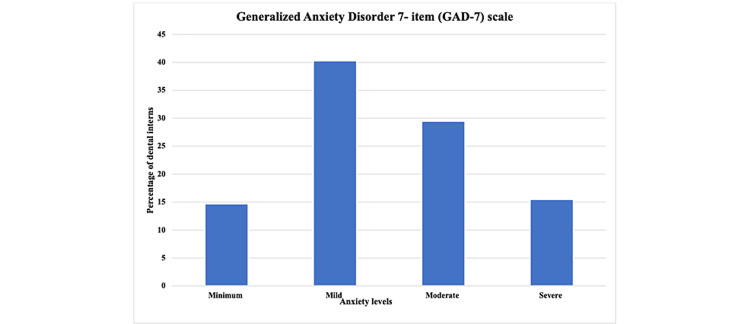
Bar chart of percentages of anxiety levels among dental interns according to GAD-7 scale categories. GAD-7 = Generalized Anxiety Disorder 7-Item

The total score for each participant was categorized, and the higher percentage was in the mild category (40.3%), as presented in Table 4 and Figure 4.

**Table 4:** Levels of anxiety GAD-7 categories. GAD-7 = Generalized Anxiety Disorder 7-Item

Severity level	Minimum anxiety	Mild anxiety	Moderate anxiety	Severe anxiety	
Frequency	19	52	38	20	Mean ± SD = 9.379 ± 4.935
Percent	14.7	40.3	29.5	15.5	P-value = 0.001*

Univariate analyses showed that females’ perceived positive satisfaction with their current social life and optimism about life were significantly associated with mild-to-moderate levels of anxiety (Table 5).

**Table 5:** Association of sociodemographic factors and GAD-7 anxiety levels. Based on Pearson’s chi-square test. P < 0.05 denotes a significant association. GAD-7 = Generalized Anxiety Disorder 7-Item

Variables	Category	GAD-7 anxiety categories.				Total	P-value
		Minimum	Mild	Moderate	Severe		
Gender	Male	7	14	5	1	27	0.02*
	Female	12	38	33	19	102	
Marital status	Married	3	3	5	1	12	0.424
	Unmarried	16	49	33	19	117	
Living status	Alone	1	2	5	2	10	0.712
	With family	15	43	30	16	104	
	In a shared house	3	7	3	2	15	
Perceived safety with COVID-19	Safe	18	48	33	15	114	0.203
	Unsafe	1	4	5	5	15	
Current country of residence	Egypt	16	45	31	20	112	0.092
	Saudi Arabia	3	7	7	0	17	
Systemic comorbidities	With systemic condition	0	3	1	3	7	0.15
	Free from any systemic condition	19	49	37	17	122	
Smoking status	Non-smoker	16	46	35	17	114	0.33
	Smoker	1	5	3	3	12	
	Quitted	2	1	0	0	3	
Perceived current social life	Satisfied	18	47	31	10	106	0.01*
	Dissatisfied	1	5	7	10	23	
Feel positive about life	Always positive	10	20	5	4	39	0.01*
	Never	0	1	0	7	8	
	Quite often	9	31	33	9	82	

The average rate of personal worries was 6.6 ± 1.05 on a scale from 1 to 10. Table 6 shows that a higher proportion of the participants significantly agreed that their profession would be risky (87.6%), their capability to do their job to the best would be jeopardized (64.34%), and they would need extra protections in the clinical training post-COVID-19 (89.92%). They disagreed with postponing their training until COVID-19 cases declined entirely (75.97%). Most had no thoughts of better being off dead or hurting themselves (77.51%), trying to kill themselves, or attempting suicide (88.37%).

**Table 6:** Future subjective worries.

Subjective worry questions	Response, n (%)		Mean scale grading (0–10) ± SD	P-value
	Yes	No		
Will your profession be risky post-COVID-19?	113 (87.6%)	16 (12.4%)	7.5 ± 1.95	0.001*
Will your ability to perform your job to the best be jeopardized post-COVID-19?	83 (64.34%)	46 (35.66%)	7.15 ± 1.8	0.001*
Do you prefer to postpone your training until COVID-19 cases decline completely?	31 (24.03%)	98 (75.97%)	5.98 ± 2.72	0.001*
Will there be a need for extra precautions in clinical training after COVID-19 cases decline?	116 (89.92%)	13 (10.07%)	7.52 ± 1.94	0.001*
Would you choose another profession if given a chance?	66 (51.16%)	63 (48.83%)	7.45 ± 2.42	0.793
Do you have thoughts that you would be better off dead or hurting yourself in some way?	29 (22.48%)	100 (77.51%)	5.68 ± 2.79	0.001*
Have you ever in your whole life tried to kill yourself or made a suicide attempt?	15 (11.62%)	114 (88.37%)	4.97 ± 2.9	0.001*

Table 7 presents the association of the personal worries of the participants to the psychological distress K-10 levels and anxiety GAD-7 levels. A significant association was reported only with the disagreement that they would better be off dead or hurting themselves in any way with high levels of psychological distress (p = 0.005).

**Table 7:** Association of future subjective worries to K-10 and GAD-7 categories. K-10 = Kessler Psychological Distress Scale; GAD-7 = Generalized Anxiety Disorder 7-Item

Subjective worry questions	Levels of psychological distress (K-10) categories						Levels of anxiety (GAD-7) categories					
		Low	Moderate	High	Very high	P value		Minimum	Mild	Moderate	Severe	P-value
Will your profession be risky post-COVID-19?	No	0	3	7	6	0.618	No	5	4	6	1	0.137
	Yes	3	31	36	43		Yes	14	48	32	19	
Will your ability to perform your job to the best be jeopardized post-COVID-19?	No	0	12	16	18	0.437	No	8	16	14	8	0.783
	Yes	3	22	27	31		Yes	11	36	24	12	
Do you prefer to postpone your training until COVID-19 cases decline completely?	No	3	28	31	36	0.397	No	15	42	26	15	0.591
	Yes	0	6	12	13		Yes	4	10	12	5	
Will there be a need for extra precautions in clinical training after COVID-19 cases decline?	No	1	4	6	2	0.222	No	2	7	2	2	0.623
	Yes	2	30	37	47		Yes	17	45	36	18	
Would you choose another profession if given a chance?	No	3	17	24	19	0.067	No	10	30	16	7	0.265
	Yes	0	17	19	30		Yes	9	22	22	13	
Do you have thoughts that you would be better off dead or hurting yourself in some way?	No	3	30	37	30	0.005*	No	18	41	30	11	0.25
	Yes	0	4	6	19		Yes	1	11	8	9	
Have you ever in your whole life tried to kill yourself or made a suicide attempt?	No	3	31	41	39	0.083	No	19	47	31	17	0.086
	Yes	0	3	2	10		Yes	0	5	7	3	

## Discussion

Stress, anxiety, and personal worries are common responses to a challenging physical or psychological condition, which could negatively impact mental and physical health and hinder a person from performing their usual daily activities or impede them from moving on with their current life. Dental learning is usually considered a tense learning environment [15]. The COVID-19 outbreak, its associated confinement, and continuously emerging variants, dental practice and fear of infection are among dental students’ reported stressors, which can lead to psychological problems [13,16]. More evidence needs to be described related to the likely effect of the pandemic on higher education in Middle Eastern dental students. Accordingly, this study investigated the hypothesis that dental students could have raised stress and anxiety levels post-COVID-19, which may impact their future dental profession.

This study investigated the psychological impact of COVID-19 in two dental governmental colleges in two countries considering earlier studies’ recommendations to conduct a cross-sectional survey at different dental schools [17]. Students at different schools with different online experiences might respond differently. In addition, this study was conducted after the pandemic and returning to everyday life, which differs from most reported research conducted during the active phase of the pandemic [3,8,9,11] or shortly after [14]. These studies assessed psychological changes related to the sudden transformations in the students’ lives. They could not evaluate the accumulative effect of the lockdown and the suspension of all aspects of life for extended periods.

In this study, the K-10 psychological distress scale prevailed (71.3%) in the high to very high levels among the study participants. The K-10 scale was used by Sabrina et al. [14] on a population of Bangladeshi dental students where most (84.2%) showed moderate-to-high levels of psychological distress. Current results are comparable to Alkawari and Aljabaa in Riyadh [3] and Ali et al. at Dammam [10] in Saudi Arabia, where dental students showed severe stress levels compared to assistants and faculty members, especially seniors, as well as studies carried out in Bucharest [12] and Turkey [18]. The higher stress levels in fresh graduate dental interns could be due to their break from clinical practice during the lockdown and thus missing the development of appropriate clinical skills, not receiving as good theoretical teaching as before together with worries about not becoming a good competent dentist after graduation [19]. These results are divergent from Khanagar and Alfadley [9], who reported COVID-19 psychological stress in 0.9% of the dental interns, and differ from Braz-Jose et al. [20] who reported mild stress levels in dental students during the pandemic.

In this study, the GAD-7 anxiety scale showed a higher percentage in the mild level (40.3%), predominantly in females. These results differ from another study that applied the GAD-7 scale to assess anxiety in dental students but reported moderate and severe anxiety in 17% and 4% of students, respectively, with a non-significant association with gender [11]. Yildirim and Atas [8] reported high anxiety scores in Turkish dental students during the pandemic that were significantly higher in females who were assessed using the Health Anxiety Inventory (HAI), Beck Anxiety Inventory (BAI), State-Trait Anxiety Inventory-I (STAI-I), and State-Trait Anxiety Inventory II (STAI-II) questionnaires. In contrast to the study on Saudi dental interns in Riyadh at the peak of the pandemic, the prevalence of anxiety was just 7.3%. These scores were comparatively lower than the results of studies reported in the literature [9].

Despite the diverse levels of stress and anxiety reported by the participants, the mean scale of personal worries was average, reflecting usual fears regarding practicing dentistry with the coexistence of the pandemic. Most believed that their career might be risky and that they would need extra precautions to avoid jeopardizing their careers, but they disagreed regarding postponing their training. Nevertheless, some might think of shifting careers despite insignificant. Luckily, they never thought of hurting themselves or tried to commit self-destruction. These results differed from Lingawi and Afifi [11], who reported that the majority of students (53.9%) thought that they would be able to do their job to the best of their ability post-COVID-19, 69.0% of students did not wish to change their career if given a chance, and there was a significant relation between anxiety level and students’ response to their need for extra precautions during dental training. In contrast, according to Hattar et al. [17], the fifth-year dental students at Jordan University who were affected by changing the learning face-to-face strategy to a distant one during the pandemic reported an overall promising self-perceived preparedness level, despite the fact they had doubts regarding independent practice following graduation. Usually, younger practitioners express more concerns about their future than older pre-doctoral students but respond more positively about adjustments [21].

Limitations of the current study included being an online survey which may show bias [3]. Students who were active online and had good internet connections were more likely to respond to this study. On the other hand, anxious students might respond more likely to psychological surveys, which might result in selection bias [14]. A further limitation was that students from all academic years were not included in the study. The first- to third-year students primarily study basic sciences and practice dental skills in phantom labs, reducing the impact of virtual transformation [6]. Thus, pre-clinical students were not required to contact patients and were not subjected to the same levels of worry to develop their manual skills [14,22]. Nevertheless, this study was the first of its kind in Egypt compared to their Saudi Arabian counterparts to reveal dental intern students’ psychological distress, fear, and coping strategies.

## Conclusions

Dental interns experienced high psychological distress and moderate anxiety levels in response to practicing dentistry post-COVID-19 pandemic. In addition, females were more stressed than their counterparts. The participants reported average worries concerning the future practice of the dental profession and believed that they should not postpone their training or shift their careers but needed to apply extra precautions to avoid jeopardizing their careers. This study recommends regular checkups of students’ stress levels and evolving policies and support approaches to address the health and well-being of dental students besides the core support of academic and clinical skills development.
